# Communicating in Palliative Care for Neurodegenerative Diseases: A Qualitative Study on Professional–Family Interactions

**DOI:** 10.3390/brainsci16050481

**Published:** 2026-04-29

**Authors:** Barbara Rizzi, Maria Chiara Gandini, Andreina Saba, Giada Lonati, Angela Recchia

**Affiliations:** 1 Fondazione VIDAS ETS, Via Ugo Ojetti 66, 20151 Milano, Italy; barbara.rizzi@vidas.it; 2 VIDAS ODV, Via Ugo Ojetti 66, 20151 Milano, Italy; chiara.gandini@vidas.it (M.C.G.); giada.lonati@vidas.it (G.L.); 3 Department of Primary Care, Azienda USL-IRCCS of Reggio Emilia, 42122 Reggio Emilia, Italy; andreina.saba@ausl.re.it

**Keywords:** palliative care, neurodegenerative diseases, communication, health care professional, family, caregiver, qualitative research

## Abstract

**Highlights:**

**What are the main findings?**

- A dedicated palliative care approach is required for families of patients with neurodegenerative disorders, particularly when patients lose decision making capacity and clinicians should guide families through critical choices in the absence of advance care planning (ACP).
- A palliative care paradigm shift is needed, strengthening professionals’ competencies in neurodegenerative diseases and promoting closer collaboration across care health care providers to improve early patient and family knowledge of the disease evolution.

**What are the implications of the main findings?**

- Advancing reflections within palliative care to identify key elements for a paradigm shift and strengthen collaboration among all health care providers across the neurological disease trajectory.
- Recognizing the patient–caregiver dyad as central to neuropalliative care, with attention to caregiver vulnerability and the need for psychological and emotional support at end of life.

**Abstract:**

**Background/Objectives**: In Palliative Care (PC), the communication is an essential aspect of care becoming particularly significant at the end-of-life. In neurodegenerative diseases, communication involves additional complexity due to prolonged disease trajectories, early cognitive decline, and frequent loss of decision-making capacity. The aim of this study was to explore PC healthcare professionals’ experiences with communication process and relational dynamics involving families of patients with advanced and terminal neurogenerative disease. **Methods**: The study design was qualitative, using semi-structured interviews and reflexive thematic analysis. Participants were healthcare professionals directly involved in communication with the family. **Results**: Twenty PC professionals were interviewed, generating 792 coded excerpts. Four themes emerged: (1) Navigating PC in neurodegenerative diseases, highlighting shift from oncology-centred palliative models toward neuropalliative care, with distinctive relational challenges; (2) Navigating conversations between professionals and families, describing multidisciplinary communication, core clinical and emotional topics, and goal-oriented decision-making in contexts of impaired patient capacity; (3) Facing challenges in health care professional–family communication, including conspiracy of silence, absence of Advance Treatment Directives (ATD) or Shared Care Planning (SCP), and limited collaboration with neurologists; and (4) Envisioning methods for improvement, emphasizing the need for disease-specific competencies, advanced relational skills, interprofessional coordination, and support for professionals’ emotional wellbeing. **Conclusions**: Communication in neurodegenerative palliative care is an ongoing relational and interpretative process requiring professionals to mediate uncertainty, surrogate decision-making, and caregiver burden. Strengthening disease-specific communication skills, early integrated PC, and structured interprofessional collaboration may enhance shared decision-making, caregiver support, and care continuity.

## 1. Introduction

Palliative Care (PC) is defined as active holistic care for individuals experiencing serious health-related suffering that cannot be relieved without medical intervention and that substantially compromises physical, social, spiritual, and/or emotional functioning; the aim of PC is to improve the quality of life of patients, their families, and their caregivers [1]. This framework is particularly relevant for patients with severe illness, that have a high mortality risk, significant impairment in daily functioning and quality of life, and heavy symptoms and treatment. Moreover, their caregiver has significant burden.

Patients affected by neurodegenerative disease, like Parkinson’s disease, progressive supranuclear palsy, multiple system atrophy, multiple sclerosis, Amyotrophic Lateral Sclerosis (ALS), dementias, and by brain tumours and stroke-related disabilities need PC delivered through complex and multidisciplinary specialist teams [2].

The consensus statements of the European Association of Palliative Care Taskforce and European Academy of Neurology highlight the integration of PC in ordinary clinical management for people with chronic and progressive neurological diseases. Moreover, the consensus review emphasised the need to strengthen the PC professionals’ skills in neuropalliative care, supporting specific training programs and organizational interdisciplinary models [3].

In PC communication is an integral part of the care process, where the professionals’, patients’, and family’s interaction becomes especially meaningful at the end-of-life, supporting the person’s dignity, care and hope [4]. In this study, communication is not conceived as a generic exchange of information, but as a goal-directed clinical process aimed at fostering shared understanding, values-based decision-making, and alignment of care with patients’ preferences in the context of advanced and terminal neurological disease.

In recent years, the literature [5,6] has highlighted the main role of communication between healthcare professionals and the patient–family dyad as from the diagnostic stages. In the advanced and terminal stages of neurodegenerative diseases, communication enables Advanced Care Planning (ACP) and improves quality of care [5,6].

Furthermore, Anderson et al. [7] support that effective communication guarantees a good quality of end-of-life care and, in long-term, the well-being of the family and those involved. Effective communication in PC is fundamental to establish a bond of trust first with the patient but also with the family, improving the quality of care and the emotional well-being of all those involved.

Overall, the existing literature consistently supports the central role of communication in PC, particularly in facilitating ACP, improving end-of-life care quality, and supporting patients’ and families’ emotional well-being. Recent analytical syntheses confirm robust evidence on how clinicians initiate prognostic conversations, manage emotions, and introduce ACP; however, they also highlight that most studies focus on interactional practices in general palliative or oncology settings, with limited attention to neurological disease trajectories and professional perspectives across disciplines [8].

Within this conceptual framework, communication encompasses several interrelated dimensions, including discussions about prognosis, ACP, emotional support, and communication with surrogate decision-makers when patients’ decision-making capacity is compromised.

PC communication in interdisciplinary team of clinicians and nurses is often triggered by acute events or immediate needs. Such conversations become ethically challenging when patient’s loss of capacity necessitates surrogate decision-making in the context of family conflict. Further challenges include uncertainty about prognosis, limited familiarity with disease trajectories, and patient’s or proxy’s avoidance. Patients perceive the need to better understand the role of PC specialist [9].

In many neurodegenerative diseases, the decision-making capacity is impaired to such an extent that the involvement of family members or friends becomes essential [10]. The surrogate decision-makers, involved in end-of-life care, often experience significant anxiety, uncertainty, and a psychologically burdensome sense of responsibility [11,12]. Healthcare professionals play a key role in supporting family members, guiding them in taking decisions that respect the patients’ values and preferences [11]. However, much of this evidence is derived from studies focusing either on palliative populations or on patients’ and family members’ perspectives, often adopting a descriptive rather than process-oriented approach.

The focus of the current literature has primarily been on the perceptions, emotional experiences, and decision-making burdens of family members. Studies that delve deeper into the experiences of healthcare professionals, including those working in PC, are lacking. Emerging qualitative studies in neuropalliative contexts underline the complexity of communication when cognitive impairment, prognostic uncertainty, and family involvement intersect, yet remain limited in scope and focus on specific settings or professional groups, calling for further exploration of clinicians’ lived communicative experiences [13]. This gap specifically concerns how PC professionals experience and manage complex, goal-oriented communication processes with families of patients with advanced and terminal neurological diseases. This is particularly true for impaired decision-making capacity, prognostic uncertainty, and surrogate involvement context. These communicative dimensions informed the development of the interview guide, which was designed to achieve the aim of this study.

The aim of this study is to provide an in-depth exploration of PC healthcare professionals’ experiences, related to communication processes and relational dynamics involving families of adult patients with advanced and terminal neurogenerative diseases.

## 2. Materials and Methods

### 2.1. Study Design

This study adopted a qualitative design to deeply explore participants’ experiences. Data were gathered via semi-structured interviews and analyzed using Reflexive Thematic Analysis (RTA), following Braun and Clarke’s approach [14,15].

RTA was selected over other qualitative approaches, such as phenomenology, grounded theory, or content analysis, because it facilitates the exploration of subjective meanings and researcher reflexivity without aiming to generate theory or focusing exclusively on lived experience. This flexibility makes RTA particularly suitable for studies seeking rich, interpretative accounts within complex professional contexts, such as communication between healthcare professionals and family members of patients with advanced neurodegenerative diseases. To enhance transparency of methodological rigor, the analysis adhered to the Reflexive Thematic Analysis Reporting Guidelines (RTARG) [16], which provide a detailed framework of best practices and common pitfalls in the reporting of RTA [17].

The study was reported in accordance with the Consolidated Criteria for Reporting Qualitative Research (COREQ) checklist [18] (see Supplementary Materials, Table S1).

### 2.2. Setting and Participants

The study took place within VIDAS, a PC service in Northern Italy that provides hospice and home-based care. VIDAS offers comprehensive, 24 h a day, 7 days week assistance to adults and children with advanced and terminal diseases and their families.

Participants—clinicians, nurses, and psychologists—were recruited through purposive sampling to ensure diversity in age, gender, years of PC experience, and care setting (hospice or home care). Eligible participants were healthcare professionals directly responsible for communicating with family members of adult patients with advanced and terminal neurodegenerative diseases, and all provided written informed consent. Following Braun and Clarke’s methodological principles, no a priori sample size was predetermined [19]. All recruited professionals agreed to participate.

### 2.3. Research Team

The study was designed by a multidisciplinary team composed of five female professionals with backgrounds in Medicine (B.R. and G.L., MD), Nursing (M.C.G., RN; A.S., RN-MScN-PhD), and Statistics (A.R., PhD-level researcher). All team members were actively involved in clinical practice within PC settings and combined clinical, research, and academic roles. Two members of the research team had expertise in qualitative research, and the entire team was supported by an external consultant.

The variety of disciplinary perspectives enhanced the research process, allowing for a more nuanced and comprehensive understanding of communication dynamics between professionals and families, while the researchers’ professional involvement in PC was reflexively examined for its potential influence on data generation and interpretation.

### 2.4. Data Collection and Instrument

Data were collected between December 2025 and January 2026 through semi-structured interviews, a method suitable for generating rich and detailed qualitative data. Participants were informed about the aims of the study and subsequently recruited by the principal investigator (B.R.). Interviews were conducted by B.R. and M.C.G., both experienced in qualitative interviewing.

Interviews were conducted in Italian, either in person or online (via Microsoft Teams), according to participant’s preference. All interviews were audio-recorded, anonymized, and transcribed verbatim by M.C.G., B.R. and A.S.

The interview guide (Supplementary Materials, Table S2), developed based on clinical experience and the existing literature [9], explored four domains: topics and decisions, participants and documentation, challenges and improvements, and the professional’s perceived adequacy. In Table 1 the topics and the questions examples are reported.

### 2.5. Data Analysis

Data were analyzed using RTA as described by Braun and Clarke [14,20] following the six-phase: (a) familiarisation with the data; (b) systematic coding; (c) initial theme identification; (d) theme refinement and review; (e) theme definition and naming; and (f) writing up the results. The six phases of RTA were applied as flexible, iterative tools rather than rigid steps, allowing for continuous shift between phases.

Unlike purely descriptive approaches, RTA emphasises interpretative depth, plurality of meaning, and researcher reflexivity, rather than the extraction of predefined categories [15].

Initially, researchers familiarized themselves with the transcripts through repeated reading. Two researchers then independently coded the data and subsequently compared interpretations with the rest of the team, with an emphasis on interpretative plurality rather than on consensus [21]. Preliminary codes were organized into an initial thematic map, which was progressively refined. Themes and subthemes were defined, named, and supported with meaning units and coded excerpts. Field notes and reflexive memos were integrated throughout the process.

An external expert was consulted to enhance analytic rigor [14]. The research team systematically reviewed and refined themes and subthemes to accurately represent overarching patterns in participants’ accounts. Representative quotations were selected to illustrate each theme and subtheme.

In alignment with RTA principles, data saturation was not sought; instead, emphasis was placed on the richness and density of meanings across the dataset [19]. According to the research team, 20 interviews provided a sufficiently information-rich dataset for the development of well-supported themes. The entire analytic process was collaborative, with an emphasis on ongoing reflexivity and shared interpretation of the data. Consistent with reflexive thematic analysis principles, participant validation (member checking) was not conducted, as themes were understood as interpretative constructions generated through researcher reflexivity rather than as participant-verified findings.

### 2.6. Rigor and Reflexivity

Reflexivity was central throughout the study. Methodological rigor was ensured through credibility checks, sustained engagement, persistent observation, and peer debriefing. The research team engaged in ongoing critical reflection on their disciplinary backgrounds, clinical roles, prior experiences in PC, and underlying assumptions, explicitly considering how these positions might influence data collection, interpretation, and theme development [22]. Reflective memos were produced before, during, and after the analysis to enhance transparency and interpretative awareness. Continuous interaction with participants supported the development of trusting relationships, enriching data authenticity and interpretative depth.

### 2.7. Statistical Analysis

Descriptive statistical analyses were conducted to summarize continuous variables using the median, 25th percentile, and 75th percentile; categorical variables were summarized as percentages.

### 2.8. Ethical Consideration

Ethical Approval was sought and granted in October 2025 by Comitato Etico Territoriale Lombardia 3 (6307_15.10.2025_P_bis). Prior to taking part in the study, all professionals provided informed consent and were assured of privacy, anonymity, and voluntary involvement. Transcripts were anonymized, securely stored on encrypted institutional servers accessible only to authorized research team members, and managed in accordance with General Data Protection Regulation.

## 3. Results

A total of 20 healthcare professionals were invited to take part in interview, and all participants accepted the invitation to the interview. The median duration of interviews was 47 min and 30 s. Among 20 participants 40% (8) were physicians, 40% (8) nurses and 20% (4) psychologists. Participants’ ages ranged from 35 to 62 years, and they had a median of 9.5 years (4.5–15.75) experience of caring in PC. The healthcare professionals interviewed represented two distinct PC settings: 25% (5) hospice and 75% (15) home-based.

From the analysis of the excerpts, a total of 792 segments were identified through manual coding. Four main themes emerged, each described below along with their respective subthemes. To provide an overview of the qualitative findings, Table 2 summarizes the main themes and subthemes. The Supplementary Table S3 reports the themes, their respective subthemes, and illustrative examples of both significant and complete excerpts.

### 3.1. Theme 1 Navigating Palliative Care in Neurodegenerative Diseases

This theme highlights a clear difference between the care provided to end-of-life oncological patients and their caregivers, and the care for neurodegenerative diseases patients and their caregivers. Two subthemes were identified: 1.1 A shifting palliative care paradigm; 1.2 Relational challenges in care trajectories.

#### 3.1.1. A Shifting Palliative Care Paradigm

According to participants, the PC, originally developed within oncology, in recent years *“has expanded to include neurodegenerative diseases” (15.1)*, as the need for PC is increasingly recognized in this population as well, regardless of the care setting. This growing trend was reported by both home care professionals and those working in hospice, describing *“a truly significant increase in recent years” (11.1)*.

The main neurodegenerative diseases encountered include *“Alzheimer’s disease, Parkinson’s disease and related variants, dementias, and ALS” (15.2)*. Participants identified *“relevant differences” (3.1)*, particularly between patients with ALS—described as *“often younger and already aware” (3.6)* of their condition, with clear perspectives regarding their future—and patients with advanced dementias, typically older individuals who are *“limited in their autonomy and dependent on caregivers” (17.2)*.

Unlike oncological patients, for whom end of life is more readily acknowledged, neurodegenerative diseases are characterized by *“a very long trajectory with a gradual loss of autonomy” (13.1)*. These are often patients *“who survive with considerable resources” (17.4)*, making it difficult even for family members to *“let them go” (14.3)*.

#### 3.1.2. Relational Challenges in Care Trajectories

Relationships with caregivers of these patients also require a different approach. Family members are often *“very exhausted and experience anticipatory grief” (1.4)* due to the prolonged course of the illness and the gradual loss of autonomy—particularly in dementia—where care is frequently managed at home, sometimes *“due to a lack of facilities (…), sometimes for economic reasons, or also due to the patient’s explicit wishes” (11.14)*.

These individuals often have *“a long and particularly demanding caregiving trajectory behind them” (12.1)* and are frequently frightened by the *“impact of the disease on their lives” (19.2)*, including the changes it entails and the challenges of coping with the situation.

In some cases, family members appear closed, *“in denial, unprepared, and burdened by immense feelings of guilt” (8.2)*, with expectations that are completely different from those of professionals. Professionals may encounter *“closed and defensive communication” (10.10)*, where there is fear of what the professional might propose, and consequently a reluctance to ask questions, particularly when potential interventions are perceived as difficult to accept. Establishing a relationship of trust therefore becomes particularly challenging.

### 3.2. Theme 2 Navigating Conversations Between Professionals and Families

This theme explores: 2.1 Features of professional–family conversations; 2.2 Core topics addressed in conversations; and 2.3 Managing families of patients lacking decision-making capacity.

#### 3.2.1. Features of Professional–Family Conversations

When patients and families access PC, they encounter *“dedicated time for communication that they have never found elsewhere” (4.1)*. Conversations begin *“from the very beginning” (1.7)*, from the initial care encounter with both patient and family, and are conducted in a *“multidisciplinary manner” (6.1)*, meaning jointly *“by the physician and the nurse” (3.2)* and, in some cases, also *“by the psychologist” (12.4)*.

Prior to this meeting, family members meet with the social worker, whose important role is to explain *“what a PC service is and what is expected from a PC service” (8.8)*. Subsequently, conversations take place individually (physician or nurse), with *“a daily exchange” (3.23)* of information. Based on the patient’s needs, other professionals such as physiotherapists, speech therapists, or healthcare assistants may also be involved. Team discussions are often *“preceding communication and ongoing throughout the entire care process” (19.29)* to ensure alignment and to provide consistent, shared responses to the person.

Communication is primarily directed to the *“caregiver who lives with and assists the patient” (1.28)*. When there are *“other cohabiting family members”*, they are often involved in conversations *(3.25)*. In the absence of a primary family caregiver, *“also paid caregivers or personal care assistants are involved” (15.17)* in communication and decision-making.

Because the aim of these conversations is to *“make patients and caregivers as aware as possible” (1.15)* of the situation, professionals tend to avoid providing absolute truths, instead seeking to understand the patient’s genuine wishes in order to personalize care. Professionals seek to explore what patients and families already know about the prognosis and to accompany them in the *“challenging journey of awareness that leads to accepting deterioration” (11.9)*.

#### 3.2.2. Core Topics Addressed in Conversations

While differences between the two main disease trajectories—ALS and dementia—are acknowledged, participants did not report substantial differences in the topics addressed during conversations. These range from issues such as *“quality of life” (1.17)*—particularly in relation to percutaneous endoscopic gastrostomy (PEG) and tracheostomy—to *“worsening of clinical symptoms” (11.22)*, especially the onset of dysphagia and reduced respiratory function, as well as ethically significant topics such as *“respiratory emergency” (13.2)*, *“reformulation of pharmacological treatments” (4.12)*, *“physical management of the disease: pain and level of sedation” (19.18)*, *“onset of agitation” (16.10)*, and the consequent need for medication.

Conversations also address themes such as *“emotions” (7.9)* experienced by both families and patients, as well as *“signs of suffering” (16.12)*, which caregivers often struggle to recognize in patients.

One of the most relevant topics, particularly for caregivers, and highlighted by all participants, is *“nutrition and hydration” (7.8)*. According to participants, clinical deterioration, including loss of patient’s swallowing and the ability to eat, and his *“suspension of nutrition” (11.16)*, deeply frighten and destabilize family members, requiring *“specific support to help them understand” (14.13)*.

The topic of nutrition must be addressed *“by considering the degree of organ impairment” (16.12)* and the consequences of any decisions. Similarly, the issue of hydration is particularly difficult for family members, to the extent that *“the withdrawal of hydration and nutrition is less accepted by family members than death itself” (13.20)*. Some professionals, particularly psychologists, recognize the value of *“affective nourishment” (1.19)* that feeding and nutrition represent for family members and emphasize that considerable time is dedicated to this aspect.

#### 3.2.3. Managing Families of Patients Lacking Decision-Making Capacity

Family members experience concern about *“not understanding the patient’s needs” (2.3)*, and the patient’s decline is perceived with an *“intense emotional burden” (3.14)*. Families also struggle to accept the *“insurmountable limits of the disease” (4.18)* and the goals of PC. The complete *“loss of communicative competence” (2.24)* in the patient generates distress in family members and requires a specific approach from the team.

Different perceptions between families and professionals frequently coexist: family members follow a *“long and complex process of understanding and grieving” (4.1)*, unlike professionals, who must nonetheless *“learn to remain with families within this unready time” (11.4)*.

When it is no longer possible to ascertain the patient’s wishes, the focus shifts to *“reconstructing their biography” (6.3)* in order to understand what they would have wanted, through joint discussions involving physicians, nurses, and psychologists, while maintaining careful attention *“not to adopt a rigid stance” (9.6)*.

According to participants, reaching decisions that are at least understood by family members has a preventive value: *“understanding today why a feeding tube or artificial nutrition is not initiated reduces the risk of complicated grief” (8.11)*.

Despite differences in approaches depending on the underlying condition, the importance of building a trusting relationship with family members is consistently recognized. Communication with families of patients lacking decision-making capacity is not merely about providing information, but rather *“a process of listening, emotional expression, and reworking” (6.21)* that enables professionals to engage with family members and gain their trust.

### 3.3. Theme 3 Facing Challenges in Professional–Family Communication

This theme is articulated through three subthemes: 3.1 The complexity of communication processes; 3.2 Absence of Advance Treatment Directives (ATD) and Shared Care Planning (SCP); 3.3 Limited interprofessional collaboration with neurologists.

#### 3.3.1. The Complexity of Communication Processes

The complexity of conversations lies in the fact that communication aims to *“anticipate what is about to happen in order to prepare the caregiver” (7.6)* and to provide them with adaptive tools. Professionals are often faced with a family member *“already burdened by prior strain” (1.33)* related to the gradual loss of autonomy of their loved one: upon entering the home, this long-standing experience of fatigue is clearly perceived and also affects the professional.

Difficulties arise when attempting to establish a relationship of trust in order to achieve genuine awareness *(6.9)*, particularly in the presence of situations of *“conspiracy of silence” (1.37)*, where family members explicitly request *“not to disclose that this is PC” (4.20)*. In some cases, the family’s wishes may take the form of an almost irrational therapeutic obstinacy; when such attitudes risk compromising the patient’s well-being, *“the relational effort becomes particularly significant” (6.13)*.

This requires a delicate process in which the professional supports the family members *“in approaching and remaining grounded in the reality of disease progression” (11.13)*, helping them *“to accept and manage the cognitive decline of the patient” (15.9)*. Communication becomes even more challenging in situations where there are *“misalignments among family members” (2.35)* in decision-making, where there is a *“disconnect between rational and emotional awareness” (3.18)*, or where understanding is present but *“it is not possible to sustain specific decisions” (5.10)* (e.g., nutrition, respiration).

With the support of the psychologist, professionals also take responsibility for *“caregivers’ anxieties” (3.11)* striving to build a relationship of trust, starting from the patient’s perspective and self (8.9). After establishing a therapeutic alliance, they *“validate the emotions that the caregiver is experiencing: inadequacy and anger” (14.9)*. Appropriate communication helps to *“increase understanding among caregivers” (19.25)* and to untangle family dynamics, sometimes pre-existing.

There are also cases in which *“family members are excluding and do not accept the PC pathway” (3.14)* proposed by professionals. In such situations, it is advisable to wait while ultimately leaving the decision about the starting of PC to the family.

#### 3.3.2. Absence of Advance Treatment Directives (ATD) and Shared Care Planning (SCP)

During conversations, it is essential to elicit and consider *“the patient’s wishes” (7.11)*, as correctly interpreting these wishes, according to participants, helps caregivers avoid feelings of guilt. The presence of *“clear indications expressed in ATD or SCP” (7.14)* supports family members, who feel relieved from the burden of decision-making.

Due to their nature, neurodegenerative diseases *“leave enough time to work with the patient and caregiver on ATD and SCP” (19.15)*, and communication should aim to facilitate the expression and documentation of the patient’s wishes and preferences. However, in general, for these patients—except for some individuals with ALS—*“it is difficult to have formalized ATD” (1.23)*, as these are more often expressed as thoughts rather than formal documents.

When ATD are not available, professionals *“attempt to reconstruct the patient’s history” (18.12)*, starting from their biography; that is, *“to understand, by encouraging narration, what they used to say and what they wished for” (11.21)*, whether they had ever expressed desires or needs. Biography becomes fundamental, as it allows professionals to infer the patient’s preferences.

In the absence of advance directives, *“the family member is invested with significant responsibility” (13.6)*, and communication is oriented toward ensuring the best possible quality of life for the patient. Without ATD, the situation becomes more complex also for professionals, as *“they also take on the anxieties of the family member” (4.21)*.

#### 3.3.3. Limited Interprofessional Collaboration with Neurologists

Participants reported, overall, limited—or even absent—communication between the neurologists who had followed the patient during the chronic phase with both the patient and their family. It was noted that there is *“a difficulty among neurologists in addressing this type of communication” (6.9)*.

By omitting such discussions, neurologists leave *“the patient-family dyad in a gap of knowledge that is not healthy” (6.9)*. Even when communication has occurred, *“it has not been clear and has left open spaces and possibilities that no longer exist” (9.13)*, placing families in considerable difficulty.

According to participants, neurologists do not sufficiently address *“communication oriented toward the future, toward the end of life, and toward situations that may arise with disease progression” (10.1)*. As a result, patients and families *“lack prior communication” (14.1)* that should have been provided earlier in the disease trajectory.

Consequently, these patients often experience multiple hospital admissions across different settings but *“have never had adequate change or opportunity” (15.13)* to ask questions and receive consistent answers. It is as if *“one moves directly from treatable chronicity to death” (11.7)*, without intermediate phases of decline and without addressing the need for accompaniment during the advanced stages of the disease.

### 3.4. Theme 4 Envisioning Methods for Improvement

This theme is articulated through three subthemes: 4.1 Developing knowledge, skills, and competencies; 4.2 Strengthening relational dynamics; 4.3 Inner resources and personal well-being.

#### 3.4.1. Developing Knowledge, Skills, and Competencies

The increasing number of patients with cognitive impairment represents *“a challenge, for which it is necessary to learn how truly understand patients together with their families” (17.45)*.

Participants recognize that professional competence is a fundamental element for effective conversations and perceive the need to *“acquire specific skills” (19.54), including “in-depth knowledge of neurodegenerative diseases” (6.18)*, across the entire care team. Such competence supports the development of *“authority in communication with families” (6.18)* through a deep understanding of the content addressed in conversations. For example, professionals suggested training interventions that employ discussion-based approaches or *“the interpretation of real cases under external supervision” (11.15)*.

Alongside specific knowledge and disease-related competencies, participants also highlight the importance of abilities such as *“listening, compassion, and humanity” (10.12),* often developed through experience, training, and personal history. These abilities are described as valuable, including the capacity to *“hold together the discomfort sometimes felt in response to insistence of families (…) with tenderness and compassion toward them” (4.13),* as well as the subtle ability to *“deconstruct one’s own thinking in order to enter into that of the other” (6.20)*, recognizing that the empathic dimension has evolved into a more reasoned and reflective form of empathy.

#### 3.4.2. Strengthening Relational Dynamics

This subtheme encompasses two key dimensions: on the one hand, all aspects that may enhance communication with patients and families; on the other, the network of relationships within the service and with external services that may support communication processes.

Regarding the first dimension, participants suggest concrete strategies, such as *“observing how psychologists conduct their conversations” (20.29)* with patients and families, or the opportunity to *“engage in complex relational situations” (15.39)* through simulation and supervised practice with experts.

Concerning the internal dimension of the service, participants consider continuous multiprofessional team discussion essential to *“receive support” (2.16)*, as well as valuing the relationship with colleagues with whom conversations are conducted. It is considered important to *“participate in conversations led by psychologists” (6.16)* or to conduct conversations jointly with another professional in order to provide mutual support *(14.15)*. Indeed, participants emphasise that *“to communicate well, firstly it is important to communicate well among healthcare professionals and to reflect on the situation” (16.31)*.

Regarding to relationships beyond the services, participants highlight a *“lack of continuity between the neurologist and the PC approach” (3.18)* and stress the need to *“build shared treatment or care programs with the other specialists involved in patient’s care” (8.19)* and with general practitioners. This treatment approach should begin at the time of diagnosis, ensuring that earlier stages of care prepare the ground for a PC, to which families are often not adequately prepared.

#### 3.4.3. Inner Resources and Personal Well-Being

All the elements described for improvement contribute to enabling professionals to work more effectively and to perceive themselves as competent in their role. *“The professionals interviewed appeared competent, experienced, continuously striving for improvement, and deeply attentive to others. They have chosen to work in PC and are satisfied with their work” (FN3)*.

They are aware and daily perceive *“the immense suffering that precedes death” (10.7)*; yet, they are able to find the inner resources to recognize that their work can alleviate such suffering, and this is what *“has fascinated them from the very beginning” (11.12)*.

*“These are professionals who have developed compassion” (FN5)*—that is, they are able to welcome anger, the inability of family members to trust, and at times even rudeness, and to transform these reactions into compassion: *“and then you think that they have a loved one who is suffering and whom they see fading away day by day” (12.23)*. Beyond these inner resources—which are not explicitly described but are clearly perceived—participants also recognize the importance of *“continuous attention to their own professional well-being, in order to avoid saturation and maintain quality” (17.43)*. They seek opportunities within *“medical humanities and the arts”* to broaden their perspectives and engage in practices that support *“personal and spiritual well-being, such as writing and theatre” (18.20)*, helping them to process what *“this work leaves within them” (9.12)*.

## 4. Discussion

This study provides an in-depth exploration of PC healthcare professionals’ experiences, related to the communication processes and relational dynamics involving the family of adult patients with advanced and terminal neurogenerative disease. The findings illuminate a complex interplay of clinical, relational and ethical dimensions that shape conversations at the end of life in non-oncological settings. By explaining the perspectives of experienced PC professionals, this study contributes to a still underexplored area of the literature, extending current knowledge beyond the predominant focus on patients and their families. The analysis, structured around four interconnected themes—navigating palliative care in neurodegenerative disease, navigating conversations between professionals and families, facing challenges in professional–family communication, and envisioning methods for improvement—offers a coherent and in-depth interpretation of a phenomenon that is often acknowledged but under-researched. The heterogeneity of participants and the depth of interviews enabled the generation of a rich qualitative dataset, allowing a detailed reconstruction of the dynamics characterizing interactions between professionals and families of patients with advanced and terminal neurodegenerative diseases. This study addresses a relevant gap by capturing the experiential and interpretative dimensions of healthcare professionals’ marginal in the literature [23,24]. This apparent convergence reflects a shared professional culture within neuro-palliative care rather than a lack of complexity, while still allowing for nuanced differences in relational and emotional positioning across clinical contexts.

Participants described a clear and ongoing transition from an oncology-centred model of PC to a broader and more inclusive framework encompassing neurodegenerative diseases [25]. This shift is not merely epidemiological but deeply impacts the nature of care trajectories, communication processes, and relational dynamics. Neurodegenerative diseases are characterized by prolonged, uncertain trajectories marked by alternating phases of decline and of relative stability, often accompanied by early cognitive impairment and progressive loss of decision-making capacity [10,26]. In contrast to oncology, patients with neurodegenerative disease may lose the ability to fully understand and engage with their illness trajectory. This places families at the centre of decision-making processes, often without adequate preparation. This observation is consistent with qualitative findings in neuropalliative care showing that prognostic uncertainty, disease-related communication barriers, and threats to personhood fundamentally differentiate palliative care in neurological conditions from oncological trajectories [27].

A key finding concerns the persistence of informational gaps originating earlier in the care trajectory. Participants reported that non PC clinicians tend to focus on therapeutic options rather than on anticipatory communication about disease progression and end-of-life scenarios. As a result, PC teams frequently assume care at a stage where awareness is fragmented and expectations are misaligned. Limited interprofessional communication further exacerbates this fragmentation, hindering the development of shared goals and continuity of care [28]. This misalignment is particularly evident between professionals—who aim to foster realistic awareness—and caregivers, who may prioritise life-prolonging interventions despite limited clinical benefit and potential negative impact on quality of life [29]. Taken together, these findings indicate that communicative misalignment in neuropalliative care is not simply a deficit to be corrected, but a structurally embedded condition that redefines communication as an ongoing relational work of negotiation, repair, and shared meaning-making.

The emergence of neuropalliative care as a distinct subspecialty reinforces the need for dedicated competencies, particularly in communication, ethical decision-making, symptom management and the support of complex family systems [30,31].

This study highlights how families of patients with advanced and terminal neurodegenerative diseases present distinct characteristics compared to those assisting cancer patients. Referral to PC services often occurs late, at a stage when caregivers are already experiencing profound fatigue, emotional burden and limited preparedness for the progression of the disease. These findings extend the existing literature by suggesting that the multidimensional needs associated with non-malignant chronic conditions transform communication from a primarily informational exchange into a relational process through which meaning, expectations, and emotional responses are continuously negotiated between professionals, patients, and caregivers [32].

Participants emphasized that effective communication requires advanced relational competencies, including the ability to build trust through empathic engagement, validation of emotions and psychological support [33,34]. A particularly relevant aspect concerns the frequent mismatch between the caregiver’s rational understanding and emotional response regarding nutrition and hydration in end-of-life care when no longer clinically appropriate. While previous studies have extensively documented the central role of family caregivers, our findings add a professional perspective that highlights how this centrality is experienced as both a clinical necessity and a relational burden for clinicians themselves. This dual dimension is less visible in the caregiver-focused literature and suggests that caregiver centrality also reshapes professional roles and emotional labor [35,36].

End-of-life conversations typically occur in familiar settings such as the patient’s home or, when necessary, in hospice [37]. However, despite strong evidence supporting early integration of PC in the therapy of neurological disease, referrals remain infrequent and delayed [24,38]. Participants highlighted the importance of initiating communication at the time of PC enrolment, involving multidisciplinary teams—including psychologists—and maintaining continuity throughout the disease trajectory. Early PC integration is not only a timing issue, but a relational and epistemic challenge, especially in neurodegenerative trajectories where prognostic ambiguity persists.

The progressive loss of decision-making capacity represents a defining feature of advanced neurodegenerative diseases introducing significant ethical and relational complexity [39]. When direct communication with the patient is no longer possible, professionals rely on caregivers to reconstruct the patient’s values, preferences and life narrative. This process is inherently uncertain and emotionally demanding, often placing a considerable psychological burden on families [11,40]. The findings underscore how the absence of Advance Care Planning (ACP) further amplifies this burden. International data indicate low levels of ACP uptake—for example, only 37% of individuals in the United States engage in formal ACP [40]. The professionals interviewed confirm that ACP supports patient autonomy, yet its practical adoption remains limited, except for specific groups as ALS patients.

In the absence of ATD and in the impossibility of developing a SCP, caregivers assume a central decisional role, often without sufficient guidance. While this increases complexity for professionals, participants also highlighted their capacity to support families in navigating uncertainty and emotional distress [41]. Evidence suggests that facilitated, co-constructed SCP processes can reduce post-traumatic stress among bereaved families and improve the quality of end-of-life care [42]. These findings suggest that in neurodegenerative disease, Advance Care Planning should be understood not as a singular preparatory act, but as a dynamic, relational process shaped by prognostic uncertainty, evolving capacities, and ongoing professional–family mediation.

### 4.1. Limitations

This study is based on qualitative interviews with experienced PC professionals working with both oncological and neurodegenerative disease patients. While the depth and richness of the data provide valuable insights, the perspectives of patients and family caregivers were not included and should be explored in future research. Additionally, the findings may not be fully transferable to all PC contexts, given differences in organisational structures, and healthcare systems. The researchers’ professional involvement in PC represented both a strength and a potential limitation, as it facilitated in-depth contextual understanding while possibly influencing data interpretation; this risk was mitigated through the application of established qualitative rigor strategies.

### 4.2. Implications for Practice

The findings of this study highlight the need for a more anticipatory and integrated approach to PC in the context of advanced and terminal neurodegenerative diseases. Earlier integration may help reduce informational gaps, support a more gradual development of awareness, and enable family caregivers to better adapt to the progressive and often unpredictable course of illness.

At the same time, the findings underscore the need for specialized training in neuropalliative care. The complexity of these conditions, characterized by progressive cognitive decline and intricate family dynamics, calls for ongoing, multiprofessional training. A particularly innovative aspect of such training lies in the opportunity to discuss real-world challenging cases with the support of supervisors external to the care team.

A central implication concerns the role of communication as a shared and interprofessional process. Limited exchange of information between specialists, primary care providers and PC teams contributes to fragmented care trajectories and inconsistent messaging, ultimately increasing the burden on families. Developing coordinated communication and promoting SCP may foster greater alignment among professionals and provide patients and caregivers with more coherent and continuous support.

Within this framework, the pivotal role of family caregivers emerges clearly. Caregivers are often involved in long-term, demanding care trajectories and may reach PC services in conditions of significant emotional and physical exhaustion. This highlights the need for sustained, personalized support interventions that include psychological care, structured communication and regular follow-up. The integration of mental health professionals within PC teams appears particularly relevant in addressing the complex emotional and relational challenges identified in this study.

Furthermore, participants highlighted that the absence of formal advance directives often places caregivers in a surrogate decision-making role marked by uncertainty and emotional strain. Early and ongoing about ACP discussions across the neurodegenerative disease trajectory may help reduce decisional burden and better support families at end of life [43].

Overall, these findings suggest that improving PC for neurodegenerative conditions requires not only clinical expertise but also organizational innovation and a sustained focus on communication and relational care. Moving toward models that ensure continuity, shared decision-making and early engagement with patients and families may enhance both professional effectiveness and the well-being of those involved in care.

## 5. Conclusions

This study highlights the specific challenges encountered by PC professionals in supporting patients with advanced and terminal neurodegenerative diseases and their families. The findings add to existing knowledge by emphasizing the need for earlier integration of PC, more intentional and structured communication practices, and the development of dedicated neuropalliative competencies.

Persistent gaps in prognostic communication, late referrals, and limited ACP contribute to increased caregiver burden and hinder shared decision-making. From a practice perspective, strengthening interprofessional collaboration and adopting proactive, family-centred communication strategies may enhance continuity of care and reduce emotional distress.

Overall, the results support the ongoing evolution of PC models that more effectively address the needs of non-oncological patients. While grounded in a specific clinical and organizational context, these findings underscore the importance of investing in ACP, timely referrals and multidisciplinary approaches to improving the quality of end-of-life care for individuals with neurodegenerative diseases and their families.

## Figures and Tables

**Table 1:** Topics and examples of questions.

Topics	Questions Examples
Topics and decisions	Referring to your experience, what topics are discussed?
Participants and documentation	How is the family member involved?
Challenges and improvements	What were the biggest difficulties in meaning conversations?
Professional’s perceived adequacy	What needs do you perceive to better manage conversations?

**Table 2:** Main themes and subthemes.

Theme	Subthemes
1. Navigating palliative care in neurodegenerative diseases	1.1 A shifting palliative care paradigm
	1.2 Relational challenges in care trajectories
2. Navigating conversations between professionals and families	2.1 Features of professional–family conversations
	2.2 Core topics addressed in conversations
	2.3 Managing families of patients lacking decision-making capacity
3. Facing challenges in professional–family communication	3.1 The complexity of communication processes
	3.2 Absence of Advance Treatment Directives and Shared Care Planning
	3.3 Limited interprofessional collaboration with neurologists
4. Envisioning methods for improvement	4.1 Developing knowledge, skills, and competencies
	4.2 Strengthening relational dynamics
	4.3 Inner resources and personal well-being

## Data Availability

Data available on request due to restrictions.

## Supplementary Materials

The following supporting information can be downloaded at: https://www.mdpi.com/article/10.3390/brainsci16050481/s1. Table S1. COREQ Checklist; Table S2. Semi-structured interview; Table S3. Themes, related sub-themes, and selected illustrative excerpts.

## Abbreviations

The following abbreviations are used in this manuscript:

ACP	Advance Care Planning
ALS	Amyotrophic Lateral Sclerosis
ATD	Advance Treatment Directive
COREQ	Consolidated Criteria for Reporting Qualitative Research
PC	Palliative Care
RTA	Reflexive Thematic Analysis
SCP	Shared Care Planning
