# Supplementary material for: Communicating in Palliative Care for Neurodegenerative Diseases: A Qualitative Study on Professional–Family Interactions
Source: Brain Sci. 2026 Apr 29;16(5):481. doi: 10.3390/brainsci16050481 (PMC13204002; doi:10.3390/brainsci16050481)
Supplement: Supplementary file 1 [file brainsci-16-00481-s001.zip › Suppl File_Table S 3_Themes.pdf]

Supplementary Table S3. Integrated Themes, Sub-Themes, and Selected Codes

THEME	SUB-THEMES	SELECTED CODES
1. Navigating palliative care in neurodegenerative diseases	1.1 A shifting palliative care paradigm	*'I have been working in VIDAS for several years, and the palliative care framework has truly been a journey toward diseases that were not oncological… Over the years the gap has widened, especially in the last five years, regarding patients with neurodegenerative conditions.'* (17.1)
		'The prevalent diseases are Alzheimer's disease, Parkinson's disease and related variants, dementias, and ALS.' (15.2)
	1.2 Relational challenges in care trajectories	*'Unlike with oncological patients, communication is more complex… helping family members understand the end of life is exhausting.'* (13.1)
		'These are people with a considerable suffering burden… care provided over a very long period.' (12.1)
2. Navigating conversations between professionals and families	2.1 Features of professional-family conversations	*'We revisit elements with family members to understand their strengths and difficulties… we explain our palliative care approach.'* (3.11)
		'What we consider is truly a process… many conversations are needed to identify the patient's values.' (1.13)
	2.2 Core topics addressed in conversations	*'When we explain to a layperson that hydration's water may enter the lungs when the regulatory system no longer works… it can be more convincing.'* (4.17)

		'We try to understand whether prognosis and quality of life have been addressed and explain what the palliative approach is" (3.16)
	2.3 Managing families of patients lacking decision-making capacity	*'Family members often do not suspect patient's pain, whereas the team observes it almost in all cases… treatments are effective.'* (2.4)
		'In dementia, communication respects the patient's life history… in ALS, we help families accept the patient's wishes.' (4.2)
3. Facing challenges in professional-family communication	3.1 The complexity of communication processes	*'I perceive the fatigue - preexisting fatigue… loss of autonomy is already a form of grief.'* (1.33)
		'There are divergences among caregivers… some are aligned, others are not.' (3.20)
	3.2 Absence of Advance Treatment Directives and Shared Care Planning	*'We reconstruct the patient's story to understand his wishes.'* (7.11)
		'People are not ready for many reasons: fear, anger, cultural factors, or advanced cognitive decline.' (19.16)
	3.3 Limited interprofessional collaboration with neurologists	*'Neurologists struggle to have these conversations… leaving the patient–family dyad exposed to knowledge gaps.'* (6.9)
		'transition to palliative care is a critical delicate procedure not always shared with other clinicians.' (1.8)
4. Envisioning methods for improvement	4.1 Developing knowledge, skills, and competencies	*'Skills acquired during training and previous experience, together with humanity and compassion, are essential.'* (10.12)

	'Competence supports clear communication and conveys reliability.' (6.18)
4.2 Strengthening relational dynamics	*'Relational time is therapeutic time… interventions depend on trust and openness.'* (16.27)
	'Ideally conversations should begin when the neurologist makes the diagnosis, explaining the expected disease's course.' (8.7)
4.3 Inner resources and personal well-being	*'Professionals must reflect on what parts of themselves enter in the care relationship… we are never only our roles.'* (4.21)
