# Supplementary material for: Communicating in Palliative Care for Neurodegenerative Diseases: A Qualitative Study on Professional–Family Interactions
Source: Brain Sci. 2026 Apr 29;16(5):481. doi: 10.3390/brainsci16050481 (PMC13204002; doi:10.3390/brainsci16050481)
Supplement: Supplementary file 1 [file brainsci-16-00481-s001.zip › Table S 2_Supplementary_Interview_Guide_English.pdf]

Supplementary Material – Semi-Structured Interview Guide

Experiences of Communication Between Palliative Care Professionals and Family Members of Patients with Advanced and Terminal Neurodegenerative Diseases: A Qualitative Study

(VIDAS – Qualitative Study, Version 1.0 – 15/07/2025)

INSTRUCTIONS FOR THE INTERVIEWER: HOW TO START THE INTERVIEW

Before beginning the interview, provide information about the study, hand over the participant information sheet, and collect informed consent through the signed consent form.

The observer is responsible for monitoring and noting 'memos' related to the interviewee's nonverbal communication.

The interview aims to explore the experiences of Health Care Professionals working in a Palliative Care service regarding communication with family members of adult patients with advanced and terminal neurodegenerative diseases.

It includes four sections intended to investigate key areas:

1. Topics and Decisions

2. Participants and Documentation

3. Challenges and Improvements

4. Perceived Adequacy by the Health Care Professional

For each area, a description outlines the core elements of the questions. This serves only as guidance for the interviewer.

INSTRUCTIONS FOR THE INTERVIEWER

Thank the partecipats, make them comfortable, and provide clarifications as needed.

THE INTERVIEW

Opening Questions:

Have you had the opportunity to meet family members of patients with advanced and terminal neurodegenerative diseases (which disease most frequently)?

What is your overall perception of communication with these family members?

Theme 1: Topics and Decisions

(This section explores how and when conversations start, who initiates them, how they develop, and what topics are discussed, including Advance Treatment Directives (ATD) and Shared Care Planning (SCP).

Sample Questions:

When and how is the decision to start these conversations made?

How are these conversations initiated?

What topics are usually addressed?

How do these conversations unfold?

Are there differences when the patient cannot decide autonomously, or when ATD/SCP are present or absent?

Theme 2: Participants and Documentation

(This section explores who from the team and the family participates, and how family members are involved).

Sample Questions:

Which team members are involved?

Which family members are involved?

How is the family involved?

How is the conversation documented?

Theme 3: Challenges and Improvements

(This section explores difficulties, barriers, perceived effectiveness, and suggestions for improvement).

Sample Questions:

What is your experience during these conversations?

What difficulties have you encountered?

How have these conversations been effective or ineffective?

Can you provide an example?

How could these conversations be improved?

Theme 4: Professional's perceived adequacy
(This section explores the professional's self-perception of adequacy, strengths, barriers, and training/support needs).

Sample Questions:

What allows you to feel adequate in conducting these conversations?

What prevents you from feeling adequate?

Can you provide an example?

What training or support would help improve your skills?

Final Question
Is there anything else you would like to add?

INSTRUCTIONS FOR THE INTERVIEWER: CLOSING THE INTERVIEW

Thank the participant, conclude the interview, and ask for availability for follow-up.

'Thank you very much for your time… May I contact you again if needed?'
